# Rethinking surgical success in non-cancer operations—why patient experience must lead

**DOI:** 10.1186/s41687-025-00927-9

**Published:** 2025-07-21

**Authors:** Jacob Rosenberg, Anders Gram-Hanssen, Hugin Reistrup, Jason Joe Baker

**Affiliations:** Department of Surgery, Center for Perioperative Optimization, Herlev and Gentofte Hospital, University of Copenhagen, Copenhagen, Denmark

**Keywords:** Patient-reported outcomes, Hernia surgery, Surgical evaluation, Quality of life, Patient satisfaction

## Abstract

**Background:**

Traditionally, surgical success in non-cancer operations, such as elective hernia repair, has been defined by clinical outcomes, including recurrence and complication rates. However, these measures do not capture the primary reason patients seek surgery: relief from symptoms and an improved quality of life. Despite the evident patient-centered goal of non-cancer surgical procedures, research has long prioritized clinical parameters over patient-reported outcomes (PROs). A shift is essential to ensure that surgical success aligns with what truly matters to patients.

**Main body:**

Current surgical research and practice heavily rely on clinical benchmarks that do not adequately reflect patients’ lived experiences. For non-cancer conditions, where surgery is elective and aims to enhance quality of life, PROs should serve as the primary indicators of success. Studies across various surgical disciplines have revealed discrepancies between clinical outcome measures and patient satisfaction, highlighting the need for validated, standardized PRO instruments. The Danish AFTERHERNIA Project exemplifies efforts to integrate PROs into surgical evaluations, utilizing digital health infrastructure to systematically capture patient experiences. Additionally, condition-specific tools, such as the Abdominal Hernia-Q, demonstrate the growing recognition of patient-centered metrics. However, the widespread implementation of PRO measurement faces challenges, including resource constraints and the need for clinician training. Addressing these barriers is important for redefining success in non-cancer surgical care.

**Conclusions:**

A paradigm shift in non-cancer surgical evaluation is important. Success should be measured not only by technical outcomes but also by enhancements in patient-reported quality of life and satisfaction. Incorporating PROs into surgical research and practice is both a scientific necessity and an ethical responsibility to ensure that patient needs are addressed. Moving forward, the patient experience must become the foundation for defining surgical success in non-cancer conditions. Ultimately, the primary objective of surgical intervention should be to improve the patient’s health status and overall well-being compared to their preoperative condition.

## Background

If a patient undergoes surgery for cancer, the primary concern is to be cured, and only secondarily do they consider function and quality of life. However, this is different in non-cancer surgery that is not life-threatening. In such cases, the goal is specifically to improve quality of life, and it seems quite odd that we do not measure this outcome and instead focus solely on ”hard” parameters like recurrences and infections. This, of course, should change.

When rethinking surgical success for non-cancer conditions, elective groin or ventral hernia surgery is a prime example. As a non-cancer condition, the main reason for surgery is patient-reported symptoms, also meaning that we most often do not operate if there are no symptoms; however, traditional success measures concentrate on recurrence rates and complications rather than on patients’ actual feelings postoperatively. Patients experience symptoms such as pain, bulging, or even sexual dysfunction due to the hernia, and our goal is to enhance their quality of life through hernia repair. Thus, it would be natural to ask the patient postoperatively if these symptoms have improved. This scenario also applies to other non-cancer surgical procedures, such as joint replacements, where improvements in patient-reported outcomes best define success. Nevertheless, despite this clear patient-centered objective, surgical research has long favored other outcome measures that do not adequately capture the patient’s lived experience. This must change.

In surgical research, we have long prioritized clinical parameters such as recurrence rates, complication rates, and hospital stays as the gold standard for defining success [1, 2]. While these metrics are undoubtedly important for assessing the technical quality and safety of procedures, they often overlook the most fundamental aspect of surgery for non-cancer disease: the patient’s lived experience—the very reason they sought surgery in the first place. When the primary aim of surgery is to enhance the quality of life—such as in hernia repair—it logically follows that we should measure success based on patient-reported outcomes that reflect genuine improvements in daily function, symptom relief, and overall satisfaction. Yet, despite its importance, this patient-centered approach remains underutilized in surgical research and practice.

In the case of malignant diseases, where survival is the primary goal, outcomes like cancer control and recurrence rates function as relevant benchmarks. However, non-cancer conditions create a different scenario: they are non-life-threatening, and the choice to operate usually relies on patient-reported symptoms rather than immediate medical necessity. For example, a patient undergoing hernia surgery may prioritize relief from discomfort, increased mobility, or the ability to return to physical activities without restrictions. Yet, if that same patient experiences a minor recurrence postoperatively but still sees significant improvements in their daily life, should we truly consider the surgery a failure? The gap between clinical measures and patient perspectives is considerable and requires a reevaluation of our priorities in non-cancer surgical outcomes research.

Recent advances have shown the feasibility and necessity of including patient-reported outcomes in surgical evaluations. Studies across various surgical fields—from orthopedics to general surgery to plastic surgery—indicate that patient satisfaction and quality of life outcomes often differ from traditional clinical benchmarks. For instance, in orthognathic surgery, patients frequently report significant improvements in quality of life, even when clinical outcome measures suggest suboptimal results [3]. Likewise, in spine surgery, research has highlighted the lack of validated tools to adequately assess patient satisfaction, further emphasizing the need for standardized patient-reported outcome measures [4]. The challenge, however, lies not only in recognizing the importance of patient-reported outcomes but also in developing and validating the necessary tools to capture them effectively [5].

One promising initiative paving the way for patient-reported outcome integration is the AFTERHERNIA Project [6], an ambitious nationwide effort in Denmark aimed at evaluating hernia surgery success based on multidimensional patient-reported outcomes. This nationwide study includes all Danish individuals who have undergone either groin or ventral hernia surgery over the past decade. Participants will be identified through the Danish National Patient Registry and invited by electronic reach-out for patient-reported outcome data. The questionnaire responses will be combined with clinical and patient-level information from the Danish Hernia Database. In addition, the Danish National Patient Registry holds data on long-term postoperative complications, enabling researchers to examine associations between perioperative factors, patient-reported outcomes, and long-term surgical results. By utilizing the country’s advanced digital health infrastructure [7], this initiative will provide comprehensive insights into patient experiences across various surgical techniques, facilitating a necessary shift toward patient-defined success. Such efforts illustrate how systematically incorporating patient-reported outcomes can potentially reshape surgical research and clinical practice.

To achieve widespread adoption, patient-reported outcome measurement must become a routine aspect of surgical care. This requires creating standardized, validated tools tailored for specific non-cancer surgical conditions. Instruments like the Abdominal Hernia-Q [8] and the BREAST-Q [9, 10] have shown the promise of disease-specific patient-reported outcome measures, and further progress in this arena is important. Consequently, core outcome sets that center on patient-reported outcomes are currently being developed in inguinal [11] and ventral hernia surgery [12].

As we advocate for a more patient-centered approach in non-cancer surgery, we must also acknowledge the systemic barriers to implementation. Implementing PROMs in clinical practice involves tackling challenges with key lessons like managing change, keeping stakeholders engaged, and ensuring long-term success [13]. Solutions include clear communication, having experts on the team, finding champions, and using existing technology [13]. Resource allocation, time constraints, and the need for clinician training in interpreting patient-reported outcomes present challenges that require strategic solutions. The development of automated systems for electronic communication with patients—allowing responses, for example via smartphone, to be transmitted directly to the hospital—represents a logical and feasible progression. These systems can be integrated into existing electronic health record platforms and implemented at relatively low cost. The benefits of adopting patient-reported outcomes significantly outweigh these minor obstacles. By prioritizing patient experience, we not only enhance individual care but also generate more meaningful data to refine surgical techniques, inform guidelines, and ultimately improve the overall quality of surgical practice [6].

## Conclusion

It is time for a paradigm shift. While traditional surgical metrics remain important, a more comprehensive definition of success should also incorporate outcomes that reflect what matters most to patients. Incorporating patient-reported outcomes into non-cancer surgical evaluation is not merely an academic exercise—it is an ethical imperative to ensure that we genuinely serve the needs of those who entrust us with their care. Advancing surgical care for non-cancer conditions requires placing the patient experience at the center of how success is defined. Surgical interventions should ultimately lead to greater patient satisfaction and an improved quality of life compared to the preoperative state. This patient-centered perspective should form the foundation of modern non-cancer surgical practice.

## Data Availability

Not applicable.
